# Trends in direct health care costs among US adults with atherosclerotic cardiovascular disease with and without diabetes

**DOI:** 10.1186/s12933-024-02324-w

**Published:** 2024-07-08

**Authors:** Chintal H. Shah, Gregg C. Fonarow, Justin B. Echouffo-Tcheugui

**Affiliations:** 1 Department of Practice, Sciences, and Health Outcomes Research, University of Maryland School of Pharmacy, Baltimore, MD USA; 2 Ahmanson-UCLA Cardiomyopathy Center, Ronald Reagan UCLA Medical Center, Los Angeles, CA USA; 3 Department of Medicine, Johns Hopkins University, Baltimore, MD USA; 4 Welch Center for Prevention, Epidemiology, and Clinical Research, Johns Hopkins Bloomberg School of Public Health, Baltimore, MD USA

**Keywords:** Diabetes mellitus, Type 2 diabetes, Cardiovascular disease, Costs

## Abstract

**Objective:**

Population-based national data on the trends in expenditures related to coexisting atherosclerotic cardiovascular diseases (ASCVD) and diabetes is scarce. We assessed the trends in direct health care expenditures for ASCVD among individuals with and without diabetes, which can help to better define the burden of the co-occurrence of diabetes and ASCVD.

**Methods:**

We used 12-year data (2008–2019) from the US national Medical Expenditure Panel Survey including 28,144 U.S individuals aged ≥ 18 years. Using a two-part model (adjusting for demographics, comorbidities and time), we estimated mean and adjusted incremental medical expenditures by diabetes status among individuals with ASCVD. The costs were direct total health care expenditures (out-of-pocket payments and payments by private insurance, Medicaid, Medicare, and other sources) from various sources (office-based visits, hospital outpatient, emergency room, inpatient hospital, pharmacy, home health care, and other medical expenditures).

**Results:**

The total direct expenditures for individuals with ASCVD increased continuously by 30% from $14,713 (95% confidence interval (CI): $13,808–$15,619) in 2008–2009 to $19,145 (95% CI: $17,988–$20,301) in 2008–2019. Individuals with diabetes had a 1.5-fold higher mean expenditure that those without diabetes. A key driver of the observed increase in direct costs was prescription drug costs, which increased by 37% among all individuals with ASCVD. The increase in prescription drug costs was more pronounced among individuals with ASCVD and diabetes, in whom a 45% increase in costs was observed, from $5184 (95% CI: $4721–$5646) in 2008–2009 to $7501 (95% CI: $6678–$8325) in 2018–2019. Individuals with ASCVD and diabetes had $5563 (95% CI: $4643–$6483) higher direct incremental expenditures compared with those without diabetes, after adjusting for demographics and comorbidities. Among US adults with ASCVD, the estimated adjusted total direct excess medical expenditures were $42 billion per year among those with diabetes vs. those without diabetes.

**Conclusions:**

In the setting of ASCVD, diabetes is associated with significantly increased health care costs, an increase that was driven by marked increase in medication costs.

**Supplementary Information:**

The online version contains supplementary material available at 10.1186/s12933-024-02324-w.

## Introduction

Atherosclerotic cardiovascular disease (ASCVD) is associated with a substantial burden of morbidity and mortality in the US [1]. Over time, the burden of ASCVD has remained high, and its costs are heightened by the implementation of an increasing number of life-prolonging therapies [1]. Indeed, the costs related to CVD are projected to continue to increase, reaching annual figures of $818 billion for direct costs and $276 billion for indirect costs by 2030 [2]. Diabetes increases the risk of CVD by at least twofold, as compared to the general population [3]. Indeed, ASCVD are the most common complications among individuals with diabetes, including peripheral artery disease, stable angina, nonfatal myocardial infarction and stroke [4].

Managing ASCVD in combination with comorbidities such as diabetes mellitus, complicates the expenses. Hitherto, the extant studies on ASCVD-related costs have seldom examined the overall impact of comorbidities such as diabetes on the direct costs [5–10]. Furthermore, these studies have been limited to short time period, focused on a single aspect of expenses (mainly in-hospital costs) or have not always had a national reach [5–10]. The extent to which the coexistence of diabetes with ASCVD affect direct medical costs (hospitalizations, outpatient visits, and other medical services) largely remain unclear. Indeed, most prior studies predated the widespread use of novel medications with robust cardioprotective effects such as the glucagon-like peptide -1 (GLP-1) receptor agonists (GLP-1RAs), the sodium glucose co-transporter -2 (SGTL2) inhibitors and the proprotein convertase subtilisin/kexin type 9 (PCSK9) inhibitors, all of which may have contributed to the rise in costs. Overall, there is a lack of nationally representative data over a prolonged time period to reliably assess the trends in resource use among ASCVD patients in the US, including in subgroups defined by diabetes status.

Using the Medical Expenditure Panel Survey Household Component (MEPS-HC) [11], the largest nationally representative survey of medical costs the United States, we examined the changes over time in direct health care expenditures among U.S. adults with ASCVD with and without diabetes from 2008 to 2019, with the aim of assessing how the burden of diabetes has affected the care of ASCVD in the US.

## Methods

### Study population

We used data from the 2008–2019 Medical Expenditure Panel Survey (MEPS) for this investigation. The data from these surveys is provided by the Agency for Healthcare Research and Quality and is a publicly available, nationally representative panel dataset. The MEPS includes several waves of national surveys of families and individuals, their medical providers, and employers in the U.S. MEPS samples data on an average of 39,000 individuals per year to estimate the use of medical resources in the U.S. population. The MEPS sample is drawn from reporting units in the previous year’s National Health Interview Survey, a nationally representative sample (with oversampling for Black and Hispanic individuals) of the US civilian non-institutionalized population. The MEPS has a complex design consisting of clustering, stratification, and multistage and disproportionate sampling with oversampling of certain population groups to ensure representativeness. The dataset contains information on demographic and socioeconomic attributes, health insurance coverage, healthcare utilization, expenditures, sources of payment, health status and functioning, along with other healthcare related information in the non-institutionalized civilian US population. Medical use and expenditures were collected from both household respondents and their medical providers. We pooled 12-year data to ensure sufficient sample size and increase precision of our estimates.

Our study focused on adults aged at least 18 years of age, with a diagnosis of ASCVD. When available, information from both the full-year consolidated data files and the medical conditions files were utilized to determine the presence of medical conditions, thereby maximizing the sensitivity of our definitions. The medical conditions and procedures reported by the MEPS-HC related to ASCVD were recorded by an interviewer as verbatim text and then converted by professional coders to *International Classification of Disease* codes. MEPS reports the first three digits of *International Classification of Disease (ICD)*-9 codes (until 2015) and *ICD-10* codes (2016 onwards) in the household component. Respondents were included in the study based on the availability of an ASCVD diagnosis at any time during the year; with no requirement for hospital admission to be included in the study [12].

### ASCVD and diabetes status definitions

ASCVD and diabetes were defined on the basis of self-report that led to medical visits or treatment within the interview year. The self-reported conditions were transcribed and classified with *ICD-9* and ICD 10-codes. Information on each respondent is annualized, in which a calendar year is the duration of time for which information is reported in MEPS.

In brief, ASCVD was determined by the presence of any coronary heart disease, myocardial infarction or angina, stroke, or peripheral vascular disease (as per the ICD codes). Diabetes was defined based on the relevant ICD codes. The *ICD -9* and *ICD-10* codes used to define ASCVD and diabetes are detailed Supplementary Table 1.

### Outcomes

Our primary outcome of interest was the annual overall healthcare related costs. These are direct medical costs including the total direct health care expenditures for the calendar year for each individual. These overall costs were also further stratified into two mutually-exclusive contributing components corresponding to prescription drug costs and overall healthcare costs besides prescription drug costs (“medical costs”). The direct medical costs were estimated by point of service, with the following point-of-service categories used: hospital (inpatient, outpatient, and emergency department), physician (office-based visits), prescription, home health, and other (including nursing home, rehabilitation, vision, medical supplies, dental). The costs include out-of-pocket payments and payments by private insurance, Medicaid, Medicare, and other sources; medical expenditures including office-based medical provider, hospital outpatient, emergency room (ER), inpatient hospital (including zero-night stays), pharmacy, dental, home health care, and other medical expenditures reported during the calendar year. The costs were adjusted to 2019 values using the Personal Health Care Expenditure (PHCE) component of the National Health Expenditure Accounts, provided by the U.S. Department of Health and Human Services [13].

We used the 12-year pooled cross-sectional data and accounted for analytic sampling weight by dividing it by the number of years being pooled, as recommended for MEPS. We combined 12 years of data (2008–2019), as over each year these have a common variance structure necessary to ensure compatibility of our variables within the complex sample design.

### Covariates

The covariates defined on the basis of self-report included demographic and clinical variables. These included: age, sex, race/ethnicity, marital status, region of the country, insurance type, family income, calendar year and comorbidities—arthritis, asthma, high cholesterol, any cancer, chronic obstructive pulmonary disease, depression, hypertension and heart failure.

### Statistical analyses

The baseline characteristics of patients are presented by diabetes status, as absolute numbers and percentages for categorical variables.

We estimated the unadjusted mean direct medical expenditures for individuals with ASCVD overall and by diabetes status. We derived the excess costs imposed by diabetes among individuals with ASCVD, which was determined via the incremental expenditure regression-based approach [14]. In our regression model, the outcome variable was expenditure (overall, medical or prescription drug costs) and the primary independent variable was the presence of comorbid diabetes. The regression approach implemented was a two-part model wherein the first part was logistic regression, and the outcome was any expenditure (binary), and the second part was a model for which the outcome was the all-cause expenditure amount. The model used for the second part was a generalized linear model (GLM), with a log function and a distribution as determined by the modified Park test [15–17]. These procedures were implemented via the ‘twopm’ and ‘glmdiag’ STATA commands, and the marginal incremental costs across the two parts was determined via the delta method [18, 19]. The covariates in adjusted models included age, insurance coverage, sex, race/ethnicity, marital status, family income, region of the country, year, and comorbid conditions (arthritis, asthma, high cholesterol, any cancer, chronic obstructive pulmonary disease, depression, hypertension, and heart failure).

Throughout the analyses, the complex survey design of the data was considered and the appropriate analytical procedures (SURVEYFREQ, SURVEYMEANS, domain statements, and svy) were implemented [20]. The Personal Health Care Expenditure (PHCE) component of the National Health Expenditure Accounts, provided by the U.S. Department of Health and Human Services, was utilized to adjust costs to 2019 values [13].

For all the analyses, we accounted for the complex sampling design of MEPS dataset by using sampling weight, variance estimation stratum and primary sampling unit (clustering). A p value < 0.05 was considered statistically significant. All analyses were performed using SAS 9.4 and STATA MP 16.

## Results

### Characteristics of the study participants

The characteristics of US adults with ASCVD with and without diabetes during the 2008–2019 period are shown in Table 1. The final study sample consisted of 28,144 individuals with ASCVD, of which 9,599 had diabetes. Compared to individuals without diabetes, individuals with ASCVD and comorbid diabetes were older (67.30 years vs. 65.27 years), more likely to be Black (24.33% vs. 18.70%) or Hispanic (20.53% vs. 15.30%) individuals, to have a larger proportion of individuals on public insurance (54.18% vs. 43.86%) and had a higher prevalence of all comorbidities examined, except for cancer. The prevalence of diabetes among individuals with ASCVD increased from 28.37% in 2008–2009 to 32.10% in 2018–2019.

**Table 1:** Characteristics of individuals with atherosclerotic cardiovascular disease by diabetes status

Characteristics	ASCVD overall (n = 28,144)	ASCVD with diabetes (n = 9599)	ASCVD without diabetes (n = 18,545)
Sociodemographic and other characteristics, n (%)			
Male	14,197 (50.44%)	4937 (51.43%)	9260 (49.43%)
Age			
Age 18–44 years	2673 (9.50%)	419 (4.37%)	2254 (12.15%)
Age 45–65 years	10,144 (36.04%)	3594 (37.44%)	6550 (35.32%)
Age > 65 years	15,327 (54.46%)	5586 (58.19%)	9741 (52.53%)
Race/ethnicity			
Race: White	20,291 (72.10%)	6435 (67.04%)	13,856 (74.72%)
Race: Black	5803 (20.62%)	2335 (24.33%)	3468 (18.70%)
Race: Other	1416 (5.03%)	575 (5.99%)	841 (4.53%)
Race: Multiple	634 (2.25%)	254 (2.65%)	380 (2.05%)
Ethnicity: Hispanic	4809 (17.09%)	1971 (20.53%)	2838 (15.30%)
Married	13,780 (48.96%)	4701 (48.97%)	9079 (48.96%)
Year			
2008–2011	9142 (32.48%)	2976 (31.01%)	6166 (33.25%)
2012–2015	9526 (33.85%)	3346 (34.85%)	6180 (33.32%)
2016–2019	9476(33.66%)	3277 (34.14%)	6199 (33.42%)
Family income			
Poor	6306 (22.41%)	2413 (25.14%)	3893 (20.99%)
Near poor	2208 (7.85%)	825 (8.59%)	1383 (7.46%)
Low income	5001 (17.77%)	1785 (18.60%)	3216 (17.34%)
Middle income	7757 (27.56%)	2625 (27.35%)	5132 (27.67%)
High income	6872 (24.42%)	1951 (20.33%)	4921 (26.54%)
Region			
Northeast	4724 (16.79%)	1641 (17.10%)	3083 (16.62%)
West	5676 (20.17%)	1797 (18.72%)	3879 (20.92%)
Midwest	5919 (21.03%)	1910 (19.90%)	4009 (21.62%)
South	11,825 (42.02%)	4251 (44.29%)	7574 (40.84%)
Insurance coverage			
Public only	13,334 (47.38%)	5201 (54.18%)	8133 (43.86%)
Any private	12,861 (45.7%)	3927 (40.91%)	8934 (48.17%)
Uninsured	1949 (6.93%)	471 (4.91%)	1478 (7.97%)
Comorbid conditions, n (%)			
Myocardial infarction	10,146 (36.05%)	3936 (41.00%)	6210 (33.49%)
Stroke	10,823 (38.46%)	3819 (39.79%)	7004 (37.77%)
Angina	6506 (23.12%)	2451 (25.53%)	4055 (21.87%)
Heart failure	1431 (5.08%)	718 (7.48%)	713 (3.84%)
Arthritis	16,495 (58.61%)	6234 (64.94%)	10,261 (55.33%)
Chronic obstructive pulmonary disease	4424 (15.72%)	1641 (17.10%)	2783 (15.01%)
Asthma	4515 (16.04%)	1791 (18.66%)	2724 (14.69%)
High cholesterol	19,553 (69.47%)	7981 (83.14%)	11,572 (62.40%)
Cancer	6391 (22.71%)	2158 (22.48%)	4233 (22.83%)
Depression	4656 (16.54%)	1839 (19.16%)	2817 (15.19%)
Healthcare spending, mean (SE)^a^			
Overall costs	$16,310 ($242)	$21,542 ($501)	$13,946 (250)
Medical costs	$12,387 ($205)	$15,372 ($400)	$11,039 (227)
Prescription costs	$3922 ($84)	$6170 ($180)	$2907 (79)

Survey weights and procedures were utilized. See text for details on ascertainment of spending. Values adjusted to 2019 US $

*ASCVD* atherosclerotic cardiovascular disease

^a^Analyses were not adjusted for covariates

### Unadjusted costs among ASCVD individuals with and without diabetes

**Fig. 1:**
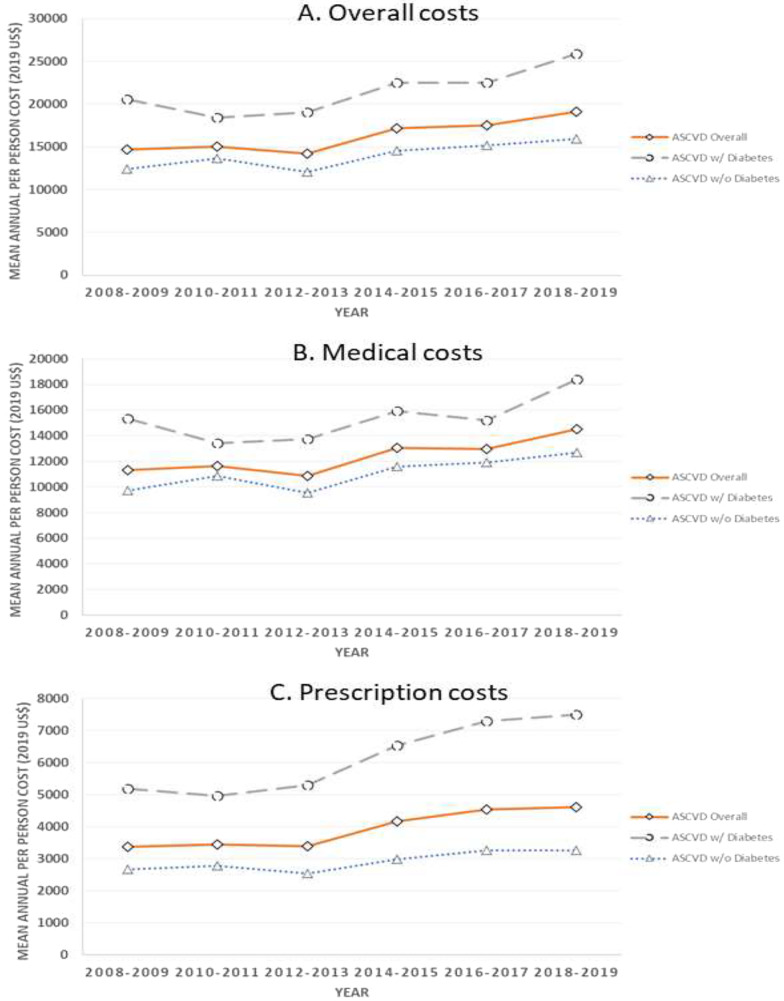
Unadjusted trends in the overall (**A**), medical (**B**), and prescription drugs (**C**) spending among persons with atherosclerotic cardiovascular disease and diabetes. Analyses were not adjusted for covariates. Survey weights and procedures were utilized

The detailed results on the unadjusted costs among individuals with ASCVD have been presented in Fig. 1 and Supplementary Tables 2, 3 and 4.

The total mean unadjusted direct expenditures for individuals with ASCVD increased continuously by 30% from $14,713 (95% confidence interval (CI): $13,808, $15,619) in 2008–2009 to $19,145 (95% CI: $17,988, $20,301) in 2018–2019 (Fig. 1A and Supplementary Table 2). There was a corresponding increase of 26% in overall cost, which was higher to begin with, among individuals with ASCVD and comorbid diabetes—from $20,539 (95% CI: $18,688, $22,389) in 2008–2009 to $25,878 (95% CI: $23,496, $28,260) in 2018–2019 through the study period (Fig. 1A and Supplementary Table 2).

The changes in medical costs among ASCVD individuals over time are shown in Fig. 1B and Supplementary Table 3. A key driver of the observed increase in costs was prescription drug costs. These drug costs increased by 37% from $3378 (95% CI: $3162, $3594) in 2008–2009 to $4618 (95% CI: $4172, $5064) in 2018–2019 among all individuals with ASCVD (Fig. 1C and Supplementary Table 4). The increase in prescription drug costs over the study period was more pronounced among individuals with ASCVD and diabetes, in whom a 45% increase in costs was observed—from $5184 (95% CI: $4721, $5646) in 2008–2009 to $7501 (95% CI: $6678, $8325) in 2018–2019 (Fig. 1C and Supplementary Table 4).

### Adjusted costs among ASCVD individuals with and without diabetes

Accounting for demographics, socio-economic factors, the effect of time and comorbidities (Table 2), the adjusted mean annual overall incremental cost related to the presence of diabetes among individuals with ASCVD was estimated to be $5563 (95% CI: $4643, $6483) based on the regression-based approach. The corresponding costs for medical care and prescription drugs were $3071 (95% CI: $2278, $3865) and $2493 (95% CI: $2238, $2748), respectively (Table 2). Prescription drug costs were responsible for almost 45% of the incremental overall cost of diabetes among these patients with ASCVD (Table 2).

**Table 2:** Incremental costs associated with diabetes among persons with atherosclerotic cardiovascular disease (ASCVD)

	ASCVD without diabetes	ASCVD with diabetes, Mean (95% confidence interval)	Specification of the models used for estimation
Overall	Reference	$5563 ($4643–$6483)	Two-part regression: first part: logit; second part: generalized linear model (family = Poisson, link = log)
Medical	Reference	$3071 ($2278–$3865)	Two-part regression: first part: logit; second part: generalized linear model (family = Poisson, link = log)
Prescription	Reference	$2493 ($2238–$2748)	Two-part regression: first part: logit; second part: generalized linear model (family = gamma, link = log)

Values in 2019 US dollars. All differences were significant at 95% confidence interval. The covariates in adjusted models included: age (10-year intervals), insurance coverage, sex, race, ethnicity, marital status, family income, region, year, and comorbid conditions (arthritis, asthma, high cholesterol, any cancer, chronic obstructive pulmonary disease, depression, hypertension, and heart failure)

*ASCVD* Atherosclerotic cardiovascular disease

### Economic burden of diabetes and ASCVD in the US

We extrapolated the individual costs estimates, to the entire US population, as shown in Supplementary Table 5. Based on the unadjusted mean, the annual aggregate cost during the 2008–2019 period among adults with ASCVD was estimated at $394 billion for the entire US population. These are unadjusted costs and represent all-cause costs among ASCVD patients. At the population level, the adjusted total incremental cost due to diabetes among individuals with ASCVD was $42 billion per year, when comparing individuals with diabetes to those without diabetes.

## Discussion

We demonstrated that direct health care expenditures among adults with ASCVD with and without diabetes increased from 2008–2009 through 2018–2019 (by ~ 30%). Individuals with diabetes and ASCVD had ~ 1.5 times higher total direct health care expenditures compared with those without diabetes during the 2008–2019-time frame. These trends may reflect a number of factors including a longer survival of individuals with ASCVD and a better management over time with an implementation of quality of care standards, which include drug prescription. Indeed, the temporal trends for increased total medical expenditures was driven largely by prescription drugs, with a relatively modest contribution for other types of expenditures. An important proportion of cardioprotective drugs are now prescribed among those with ASCVD, especially among those with diabetes. Indeed, important diabetes medications with cardioprotective effects include GLP-1RAs and SGLT2 inhibitors. Moreover, the prevalence of diabetes increased over the study period [21], which may partially explain the increase in the costs in those with comorbid diabetes. During the study period there was also a surge in the number of diabetes medications with an effect on the cardiovascular system. In our regression models, other comorbidities did not influence the ASCVD related costs to the same extent as diabetes.

Our study provides important insights into factors associated with ASCVD expenditures, thus has important implications for providing value-driven care to ASCVD patients. The observed trends can be used to evaluate the effectiveness of the CVD control and prevention programs, and point to the potential needs for a shift in the delivery of care to a more comprehensive approach to the management of ASCVD that includes concomitant management of metabolic comorbidities. The disproportionately elevated costs of ASCVD especially in the setting of diabetes points to the need for community-based efforts aimed at preventing metabolic conditions, which might affect both the occurrence and progression of ASCVD. Such preventive efforts are highly needed given the projected increases in direct or indirect costs of CVD by 2030 [2].

Our study is unique as it comprehensively examines US national trends in expenditures related to ASCVD, over a substantially long period of time (more than a decade), including data on inpatient, outpatient, ER visits and prescription medication use, and also examining subgroups by diabetes status. To our knowledge, studies have seldom or not used national level data to examine the trend of the financial burden of ASCVD from the patients’ or payers’ perspective, including prescriptions and non-prescription drug costs, and accounting for the diabetes status. Our results are consistent with those of a prior study using claims data and examining the costs of care for ASCVD among patients with and without type 2 diabetes [5]. Other prior studies on the costs of ASCVD have tended to focus on either shorter periods of time (a few years), in-patient care/hospital costs only, or the use of lipid-lowering medications only [6–10]. Furthermore, the aforementioned prior studies have mainly predated the widespread use of novel cardioprotective medication such as the PCSK9 inhibitors, SGLT2 inhibitors and GLP-1 receptor agonists, which have costs that can potentially outweighs all the other ASCVD costs. Indeed, recent data indicates very high costs related to the use of SGLT2 inhibitors and GLP-1 receptor agonists among individuals with diabetes in particular [22], as well as for PCSK9 inhibitor therapy in the overall ASCVD population [23].

The strengths of our study include the examination of trends in costs over more than a decade using a nationally representative sample, including multiple cost categories (inpatient, outpatient, prescription medications, dental, ER, and home health expenditures) and the use of a robust cost estimation method to assess incremental costs (including those related to the presence of diabetes) accounting for a variety of comorbidities and thus evaluating the independent effect of diabetes. Furthermore, the 12-year study period allows us to account for the changing landscape in the management of diabetes and ASCVD; especially the introduction of new therapeutic modalities including entirely new medication classes and changes in guidelines for the management of diabetes.

Our study had some limitations. First, comorbidities were based on self-report, thus a potential for bias. However, self-report of several comorbidities have been shown to be reliable [24]. Second, our estimates may be lower than the actual ASCVD costs, as an important proportion of people in the community have subclinical or asymptomatic CVD and/or undetected diabetes (up to 25% of individuals with diabetes in the community are undiagnosed [21]), which can impact the costs. Third, institutionalized individuals were not included in MEPS, they tend to be sicker but with a lower survival, but with potentially higher expenditures. Fourth, the costs were derived using survey data, with the possible sampling error; thus, there is a residual uncertainty in our point estimates, which is difficult to quantify. Fifth, we did not investigate the indirect costs of lost productivity from morbidity or premature mortality; these costs can be substantial [25]. Our analysis did not also examine types of ASCVD; the relative contribution of each type (coronary artery disease, stroke or peripheral arterial disease) to costs could have changed over time, given the continuous refinement in to the diagnosis and management of diagnose ASCVD conditions. Finally, we also lacked information on the contribution of costs devices used for diabetes (such as continuous glucose monitors and pumps and costs related to lifestyle intervention such as nutritional interventions) or for ASCVD (e.g., device for adaptation at home after stroke) management.

## Conclusion

This study provides insights into high burden of ASCVD-related costs in the United States over time, as well as the influence of diabetes on the high expenditures. Diabetes greatly contributes to the increase in ASCVD-related health costs in the U.S. population, indicating the potential savings from interventions to prevent and manage diabetes in those with ASCVD. Specifically, interventions directed towards preventing and managing metabolic factors, such as diabetes could have a significant impact on the trajectory of the overall ASCVD-related costs. Improved care access, systems of care, awareness on diet and physical activity, and reducing ASCVD risk factors such as diabetes are ways to minimize the substantial burden. The high costs of ASCVD related to the concomitant diabetes points to the need for a shift of care towards a more comprehensive approach including an integrated management of cardiometabolic disease.

### Supplementary Information

Supplementary file1 (DOCX 40 kb)

## Data Availability

The database used is publicly available.
