# Supplementary material for: Trends in direct health care costs among US adults with atherosclerotic cardiovascular disease with and without diabetes
Source: Cardiovasc Diabetol. 2024 Jul 8;23:238. doi: 10.1186/s12933-024-02324-w (PMC11232126; doi:10.1186/s12933-024-02324-w)
Supplement: Supplementary file 1 — Supplementary file1 (DOCX 40 kb) [file 12933_2024_2324_MOESM1_ESM.docx]

**SUPPLEMENTARY MATERIAL CONTENT**

**Supplementary Table 1:** Definitions for various conditions used for the analyses

**Supplementary Table 2:** Unadjusted trends in the mean (95% confidence interval) overall spending in persons with atherosclerotic cardiovascular disease with and without diabetes

**Supplementary Table 3:** Unadjusted trends in the mean (95% confidence interval) medical spending in persons with atherosclerotic cardiovascular disease with and without diabetes

**Supplementary Table 4:** Unadjusted trends in the mean (95% confidence interval) prescription drug spending in persons with atherosclerotic cardiovascular disease with and without diabetes

**Supplementary Table 1: Definitions for various conditions used in the analyses**

| **Condition** | **Definition** | |
| --- | --- | --- |
| Diabetes | Self-reported ever diabetes (excluding gestational diabetes) **OR** presence of ICD codes (ICD-9-CM: “250”/ ICD-10-CM: "E08","E09","E10","E11","E13"). |  |
| ASCVD | **Presence of any:**   - Coronary artery disease (self-reported ever coronary heart disease **OR** heart attack **OR** angina, **OR** presence of ICD codes (ICD-9-CM: “410”, “413”, “414”/ICD-10-CM: “I20”, “I21”, “I25”)) **OR** - Stroke (self-reported ever stroke **OR** presence of ICD codes (ICD-9-CM: “433”, ”434”, ”435”, ”436”, ”437”/ICD-10: “I63”, “G45”) **OR** - Peripheral vascular disease (presence of ICD codes (ICD-9: “440”, “443”/ICD-10-CM: “I70”, “I73”, “I79”)) |  |
| Abbreviations: ASCVD: Atherosclerotic cardiovascular disease; ICD-9-CM/ICD-10-CM: International classification of disease 9/10 clinical modification code | |  |

**Supplementary Table 2:** **Unadjusted trends in the mean (95% confidence interval) overall spending in persons with atherosclerotic cardiovascular disease with and without diabetes ^a^**

| **Year** | **ASCVD overall** | **ASCVD with diabetes** | **ASCVD without diabetes** |
| --- | --- | --- | --- |
| **2008-2009** | $14,713 ($13,808-$15,619) | $20,539 ($18,688-$22,389) | $12,406 ($11,519-$13,293) |
| **2010-2011** | $15,071 ($14,067-$16,074) | $18,405 ($16,645-$20,166) | $13,640 ($12,451-$14,829) |
| **2012-2013** | $14,241 ($13,341-$15,140) | $19,051 ($17,061-$21,042) | $12,062 ($11,132-$12,993) |
| **2014-2015** | $17,195 ($15,841-$18,548) | $22,478 ($19,802-$25,154) | $14,577 ($13,329-$15,825) |
| **2016-2017** | $17,515 ($16,340-$18,690) | $22,512 ($20,100-$24,925) | $15,177 ($13,862-$16,493) |
| **2018-2019** | $19,145 ($17,988-$20,301) | $25,878 ($23,496-$28,260) | $15,961 ($14,729-$17,194) |

Abbreviations: ASCVD: Atherosclerotic cardiovascular disease

^a^ Analyses were not adjusted for covariates. Survey weights and procedures were utilized. See text for details on ascertainment of spending and definitions of conditions

**Supplementary Table 3:** **Unadjusted trends in the mean (95% confidence interval) medical spending in persons with atherosclerotic cardiovascular disease with and without diabetes ^a^**

| **Year** | **ASCVD overall** | **ASCVD with diabetes** | **ASCVD without diabetes** |
| --- | --- | --- | --- |
| **2008-2009** | $11,335 ($10,501-$12,169) | $15,355 ($13,694-$17,016) | $9,743 ($8,879-$10,607) |
| **2010-2011** | $11,632 ($10,666-$12,598) | $13,433 ($11,805-$15,061) | $10,859 ($9,692-$12,026) |
| **2012-2013** | $10,846 ($10,021-$11,672) | $13,753 ($11,929-$15,578) | $9,530 ($8,660-$10,400) |
| **2014-2015** | $13,034 ($12,014-$14,055) | $15,942 ($14,215-$17,670) | $11,594 ($10,457-$12,730) |
| **2016-2017** | $12,970 ($11,932-$14,007) | $15,207 ($13,075-$17,338) | $11,923 ($10,802-$13,045) |
| **2018-2019** | $14,527 ($13,531-$15,523) | $18,377 ($16,344-$20,409) | $12,707 ($11,585-$13,829) |

Abbreviations: ASCVD: Atherosclerotic cardiovascular disease

^a^ Analyses were not adjusted for covariates. Survey weights and procedures were utilized. See text for details on ascertainment of spending and definitions of conditions

**Supplementary Table 4:** **Unadjusted trends in the mean (95% confidence interval) prescription drug spending in persons with atherosclerotic cardiovascular disease with and without diabetes ^a^**

| **Year** | **ASCVD overall** | **ASCVD with diabetes** | **ASCVD without diabetes** |
| --- | --- | --- | --- |
| **2008-2009** | $3,378 ($3,162-$3,594) | $5,184 ($4,721-$5,646) | $2,663 ($2,472-$2,854) |
| **2010-2011** | $3,439 ($3,239-$3,638) | $4,972 ($4,583-$5,362) | $2,781 ($2,576-$2,986) |
| **2012-2013** | $3,394 ($3,091-$3,697) | $5,298 ($4,702-$5,894) | $2,532 ($2,259-$2,805) |
| **2014-2015** | $4,160 ($3,662-$4,659) | $6,536 ($5,293-$7,778) | $2,984 ($2,627-$3,341) |
| **2016-2017** | $4,545 ($4,108-$4,983) | $7,306 ($6,429-$8,182) | $3,254 ($2,784-$3,724) |
| **2018-2019** | $4,618 ($4,172-$5,064) | $7,501 ($6,678-$8,325) | $3,255 ($2,807-$3,702) |

Abbreviations: ASCVD: Atherosclerotic cardiovascular disease

^a^ Analyses were not adjusted for covariates. Survey weights and procedures were utilized. See text for details on ascertainment of spending and definitions of conditions

**Supplementary Table 5:** **Extrapolated of the individual costs estimates to the entire US population with atherosclerotic cardiovascular disease with and without diabetes**

|  | **Annual cost** | **Person years for entire study period (2008-2019)** | **Aggregate cost (annual)** | **Aggregate cost for the entire study period (2008-2019)** |
| --- | --- | --- | --- | --- |
| **Unadjusted overall cost among ASCVD patients** | $16,310 | 24,164,158 | 394,117,416,980 | 4,729,409,003,760 |
| **Incremental adjusted overall cost of diabetes among ASCVD patients** | $5,563 | 7,520,119 | 41,834,421,997 | 502,013,063,964 |

Abbreviations: ASCVD: Atherosclerotic cardiovascular disease
